# Immunogenicity of RV1 and RV5 vaccines administered in standard and interchangeable mixed schedules: a randomized, double-blind, non-inferiority clinical trial in Mexican infants

**DOI:** 10.3389/fpubh.2024.1356932

**Published:** 2024-02-23

**Authors:** Mercedes Macías-Parra, Patricia Vidal-Vázquez, Jesús Reyna-Figueroa, Miguel Ángel Rodríguez-Weber, Hortensia Moreno-Macías, Inés Hernández-Benavides, Sofía Fortes-Gutiérrez, Vesta Louise Richardson, Paola Vázquez-Cárdenas

**Affiliations:** ^1^Dirección General, Instituto Nacional de Pediatría, Mexico City, Mexico; ^2^Subdirección de Investigación Biomédica, Hospital General Dr. Manuel Gea González, Mexico City, Mexico; ^3^Unidad de Enfermedades Infecciosas y Epidemiología, Instituto Nacional de Perinatología, Mexico City, Mexico; ^4^Unidad de Investigación Clínica, Instituto Nacional de Pediatría, Mexico City, Mexico; ^5^Universidad Autónoma Metropolitana, Mexico City, Mexico; ^6^Coordinación del Servicio de Guardería para el Desarrollo Integral Infantil, Dirección de Prestaciones Económicas y Sociales, Instituto Mexicano del Seguro Social, Mexico City, Mexico

**Keywords:** rotavirus, vaccine interchangeability, RV1, RV5, immunogenicity, safety, mixed schedules

## Abstract

**Introduction:**

Rotavirus-associated diarrheal diseases significantly burden healthcare systems, particularly affecting infants under five years. Both Rotarix™ (RV1) and RotaTeq™ (RV5) vaccines have been effective but have distinct application schedules and limited interchangeability data. This study aims to provide evidence on the immunogenicity, reactogenicity, and safety of mixed RV1-RV5 schedules compared to their standard counterparts.

**Methods:**

This randomized, double-blind study evaluated the non-inferiority in terms of immunogenicity of mixed rotavirus vaccine schedules compared to standard RV1 and RV5 schedules in a cohort of 1,498 healthy infants aged 6 to 10 weeks. Participants were randomly assigned to one of seven groups receiving various combinations of RV1, and RV5. Standard RV1 and RV5 schedules served as controls of immunogenicity, reactogenicity, and safety analysis. IgA antibody levels were measured from blood samples collected before the first dose and one month after the third dose. Non-inferiority was concluded if the reduction in seroresponse rate in the mixed schemes, compared to the standard highest responding scheme, did not exceed the non-inferiority margin of −0.10. Reactogenicity traits and adverse events were monitored for 30 days after each vaccination and analyzed on the entire cohort.

**Results:**

Out of the initial cohort, 1,365 infants completed the study. Immunogenicity analysis included 1,014 infants, considering IgA antibody titers ≥20 U/mL as seropositive. Mixed vaccine schedules demonstrated non-inferiority to standard schedules, with no significant differences in immunogenic response. Safety profiles were comparable across all groups, with no increased incidence of serious adverse events or intussusception.

**Conclusion:**

The study confirms that mixed rotavirus vaccine schedules are non-inferior to standard RV1 and RV5 regimens in terms of immunogenicity and safety. This finding supports the flexibility of rotavirus vaccination strategies, particularly in contexts of vaccine shortage or logistic constraints. These results contribute to the global effort to optimize rotavirus vaccination programs for broader and more effective pediatric coverage.

**Clinical trial registration**: ClinicalTrials.gov, NCT02193061.

## Introduction

Rotavirus is the leading cause of severe gastroenteritis in children below five years, responsible for almost 40% of hospitalizations in this age group. Despite a global decline in rotavirus-associated mortality following vaccine introduction, a substantial burden remains, with 129,000 deaths among children under five occurring globally in 2016 (1). From 2016 to 2019, the trend in deaths associated with rotavirus infection demonstrated a decline and exhibited a negative correlation with the sociodemographic index; regions with higher sociodemographic indexes experienced a lower burden (2). To prevent rotavirus infection, the World Health Organization (WHO) initially recommended the inclusion of the rotavirus vaccine in national immunization programs in Europe and the Americas in 2006, extending the recommendation worldwide in 2009 (3).

In regions like Latin America and the Caribbean, the implementation of rotavirus vaccines has been particularly impactful (4). In Brazil and Mexico, as two of the most populous countries, have witnessed substantial benefits from the implementation of universal rotavirus vaccination. These benefits manifest in decrease in hospital admissions, outpatient visits, cases of severe dehydration, and, most critically, in reducing diarrhea-related deaths among children under the age of five, ranging from 22 to 50% (5–8).

The efficacy of the two licensed rotavirus vaccines, RotaTeq™ (RV5) and Rotarix™ (RV1), in combating severe rotavirus-related illness has been well-established through various studies. Clinical trials and observational studies in high-income settings have demonstrated that these oral rotavirus vaccines offer more than 90% efficacy against severe rotavirus gastroenteritis in infants (9). In middle-income settings, including regions of Latin America, the efficacy ranges from 72 to 83% (10, 11). This variation in efficacy between different settings is apparent for RV1 and RV5 (11).

A comprehensive review, incorporating data from 24 countries post-vaccine licensure, reported that the median effectiveness of RV1 ranged from 57 to 84% across regions with varying child mortality rates. In contrast, RV5’s effectiveness was noted at 90% in low-mortality settings but dropped to 45% in high-mortality ones. This variation underscores the need for context-specific vaccination strategies (12).

Reduced immunogenicity of vaccines and diminished protection from natural infection are primary factors compromising efficacy in challenging settings (13). In high-income countries, seroconversion frequencies for serum anti-rotavirus immunoglobulin A (IgA) with RV1 and RV5 vaccines typically range between 87 and 95%. However, these rates are notably lower in other settings, with figures being 34.2–63.9% for RV1 (14) and 93.2% for RV5 (15). Consequently, the potential benefits of the introduction of a third dose in low-income countries, which usually follow a 2-dose RV1 schedule, have been proposed to enhance vaccine immunogenicity (11, 16).

Beyond RV1 and RV5, newer rotavirus vaccines like ROTAVAC™ (Bharat Biotech, Hyderabad, India) and RotaSIIL™ (Serum Institute of India, Pune, India), both with a three-dose schedule, have received WHO prequalification (17). These vaccines are expected to scale up globally, improving certain aspects of availability, stability, reduced cold chain requirements, and costs (17). Efficacy against severe rotavirus gastroenteritis in the first year of life for these vaccines has been reported in countries such as India (18) and Nigeria (19). Notably, a recent study from India based on a non-inferiority analysis of immunogenicity and a comparative safety profile indicates that ROTAVAC™ and RotaSIIL™ might be used interchangeably (20).

In Mexico and some Latin American countries, only RotaTeq™ (RV5) and Rotarix™ (RV1) are available through the national immunization program. RV1 was used from 2006 to 2011, succeeded by RV5 since 2011. In 2019, RV1 was partially re-introduced (21). These vaccines differ in composition and administration schedules: RV5, a pentavalent, live human-bovine reassortant oral vaccine covering serotypes G1, G2, G3, G4, and P8, is given in three-dose at 2, 4, and 6 months of age. Conversely, RV1 is a monovalent live attenuated human oral vaccine with the G1P[8] serotype, administered in a two-dose series at 2 and 4 months of age (22).

The existence of two vaccines with distinct characteristics, technical requirements, and schedules allows for the possibility of infants receiving a combination of both, especially in regions with limited supply. This is crucial when completing a vaccination schedule with just one vaccine is impractical due to various constraints. However, international recommendations on vaccine interchangeability are not consistent. For instance, Australia’s NPS MedicineWise discourages mixing the vaccines, whereas the American Academy of Pediatrics deems RV1 and RV5 interchangeable, given specific dose conversion recommendations. This is based on a 2017 clinical trial that concluded that three mixed schedules were not inferior to single RV1 and RV5 schedules in terms of immunogenicity and safety in a high-income country with very low child mortality rate (23).

The extrapolation of RV1 and RV5 vaccine interchangeability requires important considerations. In Mexico and other Latin American countries, responses to oral vaccines are often less robust compared to those observed in children from high-income settings (11). The immune response and efficacy of these vaccines are dose-dependent, and host or environmental factors may influence the effect of vaccine doses, potentially leading to reduced immunogenicity (13). Further research is necessary to determine vaccine interchangeability in various settings, considering comparative immunogenicity and safety profiles. Our study aims to evaluate the non-inferiority of five mixed vaccination schedules in terms of immunogenicity compared to the standard administration of RV1 and RV5, as well as to assess the reactogenicity and safety profiles of these schemes in Mexican infants.

## Materials and methods

### Trial design and participants

We conducted a randomized, double-blinded study (blinding of parents and observers) from November 2013 to July 2015, testing the hypothesis of non-inferiority in immune seroresponse rate and geometric means concentrations (GMC) for IgA neutralizing antibodies one month post the final vaccine dose when administered in the two standard schedules and five mixed schedules.

The study was coordinated and executed in Instituto Nacional de Pediatria (INP), a tertiary children’s hospital in México City. Infants were referred from the following hospitals: Hospital General Ajusco Medio, Hospital Materno Infantil “Magdalena Contreras,” and Hospital General “Dr. Manuel Gea González.” Healthy infants who met the criteria of medical history and physical examination were considered as eligible subjects.

The study protocol was thoroughly reviewed and received approval from the Ethics in Research Review Board of the Instituto Nacional de Pediatría (INP), and the Regulatory Agency in Mexico (COFEPRIS). The protocol adhered to the principles outlined in the Declaration of Helsinki and followed the International Conference on Harmonization Good Clinical Practice guidelines. Prior to enrollment, written informed consent was obtained from either the parents or legally acceptable representatives of the participants.

Healthy infants aged 6 to 10 weeks were enrolled and randomly assigned to one of seven study groups in balanced blocks, with each group following a distinct vaccine schedule.

Exclusion criteria included a history of allergic reactions to vaccine components, gastrointestinal diseases (acute or chronic), primary or secondary immunodeficiencies, hematological-oncological disorders, immunosuppressive medication use (e.g., prednisone for ≥2 weeks), receipt of blood transfusion or blood products (immunoglobulin) within four weeks before vaccination, participation in another study within the previous 30 days, or a gestational age of less than 37 weeks.

Infants presenting with acute illness, a rectal temperature ≥ 38.0°C within 24 h before vaccination, acute diarrhea, or antibiotic administration within 3 days prior to vaccination were rescheduled. Similarly, those who had received other vaccines within the last 28 days before the study were also rescheduled.

### Randomization and blinding

Subjects were randomized to one of seven rotavirus vaccine study groups in a 1:1 ratio in balanced blocks. The randomization codes were generated by a researcher (R-FJ) not involved in the study’s clinical procedures, data collection, or statistical analysis using the Excel function RANDBETWEEN(1,1498). These identifiers were unique to each participant, with a strict protocol in place to prevent any duplication or reassignment of numbers, ensuring the integrity of the randomization process. Access to the randomization system was limited to study personnel assigned and was used strictly in accordance with the protocol at each point of vaccine administration.

The trial employed a double-blind design, concealing the group assignments from both medical investigators and parents. Nurses responsible for administering the vaccines were the only individuals not blinded in the study.

### Interventions

This clinical trial utilized two rotavirus vaccines available in México and all Central American countries: RotaTeq™ (Merck & Co., Inc.; RV5), administered as a three-dose series at 2, 4, and 6 months of age, and Rotarix™ (GlaxoSmithKline Biologicals; RV1), given in a two-dose series at 2 and 4 months of age (5).

Rotarix™ was supplied by the National Vaccination Program, whereas RotaTeq™ was procured with funding allocated for the study. Each vaccine batch was meticulously documented by number and expiration date to ensure traceability.

The trial involved two groups that followed the standard schedules of RV1 and RV5. The other five groups received mixed schedules, combining doses of RV1 and RV5. To maintain the blinding integrity due to the differing doses between the Rotarix™ and RotaTeq™ vaccines, a placebo composed of 5% glucose solution was administered the third dose in the standard RV1 schedule group.

The specific vaccine schedules were as follows: Group 1 received RV1 + RV1 + placebo; Group 2 received RV5 + RV5 + RV5; Group 3 received RV1 + RV5 + RV5; Group 4 received RV5 + RV1 + RV1; Group 5 received RV5 + RV5 + RV1; Group 6 received RV5 + RV1 + RV5; and Group 7 received RV1 + RV5 + RV1 (Supplementary Table S1).

### Follow-up procedures

Subjects received the first vaccine dose on Day 1 (at 2 months of age ± 2 weeks), the second dose during visit 2 (at 4 months ±2 weeks), and the third dose or placebo (for Group 1) at visit 3 (at 6 months of age ± 2 weeks).

On day 1, pre-vaccination serum samples were collected before the administration of the vaccine and clinical history and physical examination were performed for all subjects.

Additionally, parents were provided with a diary to record temperature, diarrhea, stool characteristics, vomiting, local pain or redness, or any other symptoms experienced by the child in the month following vaccination. They were also given a thermometer and instructed on accurately recording temperatures in the diary.

At each subsequent visit, vital signs and general examination were conducted before each vaccine or placebo administration dose. Researchers reviewed the diary to assess solicited and unsolicited adverse events. Parents were encouraged to contact the study site or visit the hospital emergency department in case of any doubts, illness of the infant, or if they deemed it necessary, and to immediately report such incidents to the research team. Regular phone calls were made each month to follow up on adverse events or serious adverse events, remind parents of their next visit, or reschedule visits as necessary.

Concomitant vaccinations included the pentavalent vaccine (*Haemophilus influenzae* type b, diphtheria, tetanus toxoid, acellular pertussis, and inactivated polio [Hib/IPV/TDaP], Pentaxim; Sanofi Pasteur), administered intramuscularly in the right anterolateral thigh, and two doses of the 13-valent pneumococcal conjugate vaccine (PCV13; Prevenar 13, Pfizer Inc.), administered intramuscularly in the left anterolateral thigh. The hepatitis B vaccine was given at two and six months of age, with the influenza vaccine applied during the winter season (doses according to AAP annual recommendations).

Visit 4, was scheduled to collect the symptoms diaries and final serum sample one month following the administration of the last vaccine dose.

### Study objectives

The primary objective of this study was to evaluate the non-inferiority in immunogenicity of five mixed rotavirus vaccine schedules compared to the standard RV1 and RV5 schedules.

The secondary objective assessed and compared the reactogenicity-related symptoms and safety profiles across the different vaccination groups.

### Outcomes

The primary outcome was immunogenicity as measured by seroresponsiveness. Seroresponse was defined by the detection of an anti-rotavirus serum IgA concentration ≥ 20 U/mL (seropositive) at the post-vaccination sampling timepoint in infants with an IgA concentration < 20 U/mL (seronegative), at the pre-vaccination time point.

The non-inferiority margin was set at −0.10. We established this value following a conservative approach that considers the lower confidence interval limit from seroresponse rate comparing RV1 (72.1 to 81.9%) and RV5 (87 to 94.9%) schedules (24). The estimated difference between the RV1 and RV5 seroresponse rate is −14%, with a 95% confidence interval ranging from −20.24% to −7.76% (23). By taking the lower limit of this interval as our conservative benchmark and factoring in a preserved fraction of 50% of the comparative immunogenicity on mixed schedules, we derived a non-inferiority margin of −10%. This margin aims to retain a significant proportion of RV5’s protective effect while accommodating the variability inherent in mixed vaccination schedules with interchangeability with RV1 doses.

To establish non-inferiority, the lower limit of the 95% confidence interval for the difference in seroresponse proportion between each mixed schedule minus its corresponding standard schedule needed to be above the −0.10 threshold.

The secondary outcomes were the frequency of reactogenicity-related symptoms and safety assessments across all study groups.

### Immunogenicity assessment

Serum samples (4 mL each) were collected from participants prior to the first vaccine dose and one month after completing the vaccination schedule. The measurement of serum anti-rotavirus IgA levels was performed using the ELISA method at the Laboratory for Specialized Clinical Studies (LSCS) of Cincinnati Children’s Hospital.

The assay used was based on the assay described by Ward et al. (25, 26), which itself is a modification of the technique developed by Bishop et al. (27).

Microtiter plates were coated with purified rabbit anti-rotavirus polyclonal IgG to serve as the capture antibody. Rotavirus lysate from the WC3 strain and mock-infected lysate from the MA104 cell line were alternately added to the coated microtiter plates to assess non-specific binding. The plates were then incubated to allow for binding. Following incubation, the plates were washed with phosphate-buffered saline containing 0.05% Tween 20 (PBST) (Fisher Scientific, Pittsburgh, PA).

Reference standards, controls, and samples were diluted in a diluent composed of PBST with 1% non-fat dry milk. After another washing step, 50 μL of each reference standard, control, and serum sample were added to two wells pre-treated with either the rotavirus or mock-infected lysate. Plates were washed, and biotinylated goat anti-human IgA (Jackson Laboratories) was added, followed by another incubation period.

Post-incubation, the plates were washed, and peroxidase-conjugated avidin-biotin (Vector Laboratories, Inc., Burlingame, CA) diluted in wash buffer was added. The substrate O-phenylenediamine (OPD) (Sigma) was added after a final washing step. The reaction was halted after 30 min with 100 μL per well of 1.0 M sulfuric acid (H₂SO₄). Absorbance was read at 492 nm using a Molecular Devices SpectraMax 190 plate reader.

A four-parameter logistic regression function, implemented through SoftMax software, was used to model the standard curve. The reference standard, a human serum pool, was assigned a value of 1,000 units per milliliter (U/mL) for anti-rotavirus IgA. This standard established a range of concentrations to represent serial dilutions used for building the standard curve.

The concentration of anti-rotavirus IgA in test samples was determined by extrapolating from the OD values of sample wells to the standard curve. The assay’s lower limit of quantitation, which is the minimum concentration that can be measured reliably, was set at 7.5 U/mL for anti-rotavirus IgA.

The reported data resulting from this method has been thoroughly reviewed and audited in accordance with LSCS procedures (SOP No. 96), adhering to current Good Laboratory Practice (cGLP) and Good Clinical Practice (cGCP) guidelines.

The technical assay’s cut-off point was established at 20 U/mL IgA (28). This threshold has been used as a criterion for determining seropositive status at the individual level in vaccine clinical trials (20, 23, 29). It has also been used as evidence of a natural rotavirus infection (30, 31) and is correlated with efficacy outcomes, specifically a lower risk of gastroenteritis among vaccinated children (32–34).

### Reactogenicity and safety

Prospective surveillance was carried out following the administration of each dose of the vaccine to monitor for adverse events associated with vaccination, with comparisons made between groups. Parents or legal guardians recorded any instances of fever, diarrhea, or vomiting in a symptom diary within 30 days after each vaccine dose to monitor for symptoms related to reactogenicity. Follow-up visits were scheduled on day 60 post-vaccination, during which a medical researcher reviewed the completed symptom diaries. Fever was defined as a rectal temperature of 38.0°C or higher.

The safety analysis included any serious adverse events, intussusception, hospitalizations, or emergency visits reported throughout the study. The reactogenicity and safety of the vaccine schedules were assessed in the total vaccinated cohort, which comprised infants who received at least one dose of either of the rotavirus vaccines.

### Sample size

The sample size was calculated for this non-inferiority trial to ensure adequate power to detect a pre-specified non-inferiority margin.

The determination of the sample size assumed a minimum 80% seroresponding rate after the administration of a complete schedule. To achieve 80% power to demonstrate non-inferiority, with a one-sided 5% alpha level and the non-inferiority margin set according to clinical significance (−0.10), a sample size of 214 subjects per group is required. This calculation considers the potential for dropouts and non-compliance, ensuring that the trial is adequately powered to assess the primary outcome measures.

### Statistical methods

Demographic characteristics of the sample were summarized as count and percentage for categorical variables (sex) and median with interquartile ranges (25th to 75th percentile) for continuous variables (age, birth weight, and gestational age). These demographic variables were compared between groups using the Pearson Chi-square test and the Kruskal-Wallis equality of populations rank test for categorical and continuous variables, respectively.

We conducted a non-inferiority analysis to compare the concentrations of anti-rotavirus IgA in samples collected pre- and post-vaccination (one month after the last dose) from all sequential mixed vaccine groups against the two single vaccine reference groups (Groups 1 and 2). Groups 3 and 7 were compared with Group 1, and Groups 4, 5, and 6 with Group 2. This per-protocol analysis was limited to infants who adhered to the vaccination protocol and the specified sample collection intervals. We calculated the percentages of infants with post-vaccination IgA antibody concentrations ≥20 U/mL, along with their 95% confidence intervals (CIs), and determined the IgA geometric mean concentrations (GMC) with 95% CIs. Our study’s non-inferiority criterion hinged on the lower bound of the two-sided 95% CI for the seroresponse difference between each mixed vaccine schedule and its corresponding single vaccine reference. We set a pre-established noninferiority margin of −0.10, and a lower bound exceeding this threshold indicated non-inferiority. To evaluate immunogenicity, we used the two-sample test of proportions under the non-inferiority hypothesis (unilateral test for the difference between mixed schedule and standard reference group greater than zero).

Safety and reactogenicity analyses were conducted on the entire vaccinated cohort, which included all infants who received at least one dose of either the RV1 or RV5 vaccine. We tabulated the number and percentages of infants reporting each solicited and unsolicited adverse event. Differences in frequencies across groups were evaluated using a test of proportions as described above.

All statistical procedures were performed with a type I error threshold set at 5% (α = 0.05) using Stata version 18 for Mac, which was also utilized for the creation of graphical representations of the data.

## Results

**Figure 1:**
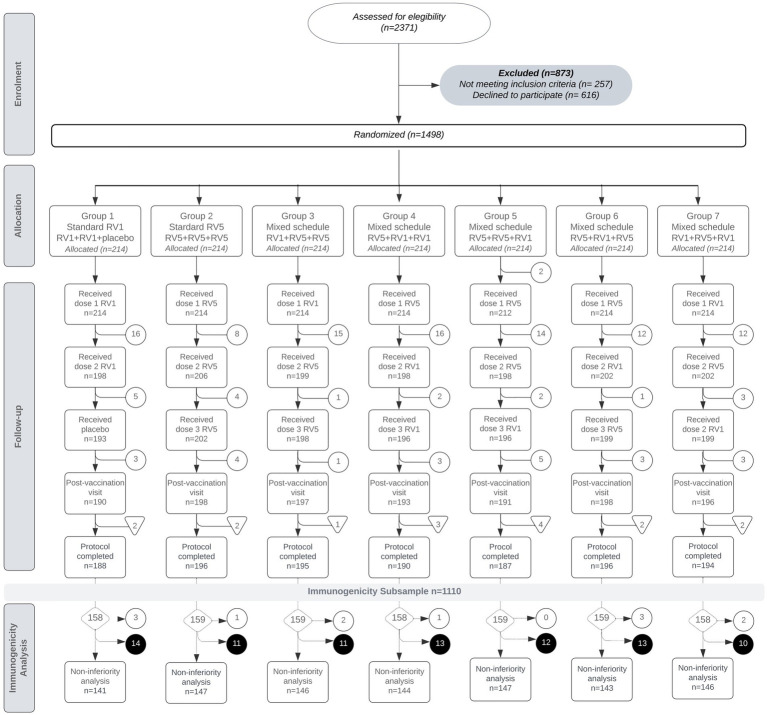
Flow diagram of participants enrollment, allocation, follow-up, and losses by group. Participant losses are marked in white circles [○]. The elimination of participants for immunogenicity analysis due to sample loss (*n* = 16) is indicated with triangles [▽] for each group. The final dataset for immunogenicity analysis (*n* = 1,110) is detailed for each group in diamonds [◇]. The elimination of subjects with IgA <20 U/mL on the pre-vaccination sample (*n* = 12) is represented in white circles [○], and subjects with missing serological data for immunogenicity analysis (*n* = 84) are denoted with black circles [●].

Between November 2013 and July 2015, 2,371 infants were assessed for eligibility. Of these, 873 were excluded: 257 did not meet inclusion criteria, and 616 declined to participate. The remaining 1,498 participants were enrolled and randomized into one of the seven groups (Table 1). Each group comprised 214 allocated participants. The participant flow and attrition at each stage of the trial were systematically documented in line to CONSORT guidelines, including the adherence to each vaccine dose and post-vaccination visits (Figure 1).

**Table 1:** Demographic characteristics of subjects enrolled.

	Group 1	Group 2	Group 3	Group 4	Group 5	Group 6	Group 7	Total sample	*p*-value*
*N*	214	214	214	214	214	214	214	1,498	
Girls, *n* (%)	100 (46.7)	122 (57.0)	106 (49.6)	104 (48.6)	104 (48.6)	109 (50.9)	111 (51.9)	756 (50.5)	0.455
Age, days	53 (47–60)	51 (46–60)	54 (48–60)	54 (48–60)	53 (46–61)	54 (47–62)	54 (47–61)	53 (47–61)	0.409
Birth weight, kg	3.11 (2.79–3.36)	3.04 (2.79–3.30)	3.10 (2.84–3.39)	3.09 (2.80–3.42)	3.07 (2.85–3.35)	3.04 (2.76–3.34)	3.06 (2.80–3.56)	3.07 (2.80–3.37)	0.585
Gestational age, weeks	39 (38–40)	39 (38–40)	39 (38.1–40)	39 (38–40)	39 (38–40)	39 (38–40)	39 (38–40)	39 (38–40)	0.890

Frequencies of female participants are presented to represent sex distribution in each intervention group. For numerical variables, medians along with the 25th and 75th percentiles (P25–P75) are provided. *The Pearson chi-square test was used for comparison of sex distribution, and the Kruskal-Wallis test was applied for comparisons of numerical variables across groups.

Group 1 received a standard RV1 regimen, concluding with a placebo dose. All participants received the first RV1 dose, 198 received the second, and 190 attended the post-vaccination visit. Immunogenicity analysis was conducted on 143 participants.

In Group 2, participants were scheduled for a standard RV5 regimen. All received the first RV5 dose; however, for one participant we did not obtain sample for pre-immunization antibody analysis, 206 received the second dose, 202 receiving the third dose, and 198 completing the post-vaccination follow-up. Immunogenicity analysis was performed on 148 subjects.

Groups 3 to 7 were assigned mixed schedules involving RV1 and RV5 doses. In the mixed groups, the retention also remained high, suggesting that the type of schedule did not significantly impact participant retention for the final analysis.

In total, 1,363 infants successfully completed the follow-up four visits. Discontinuations primarily occurred due to loss of follow-up, often related to changes in residence. Withdrawals of consent or protocol deviations, such as non-study medical interventions (e.g., blood transfusions or administering vaccine doses at non-protocol sites), were less frequent reasons for discontinuation. It is important to note that there were no cases in which the withdrawal of subjects from the study was related to the presence of adverse events attributable to the vaccines (Supplementary Table S2).

We defined the protocol complete for each participant when, in addition to attending follow-up visits, blood samples were successfully collected both pre- and post-vaccination (Figure 1).

Regarding sample loss, which led to some samples being excluded from the final analysis, the reasons included sample coagulation (*n* = 10), insufficient blood sample volume (*n* = 1), and venipuncture failure (*n* = 5) (Supplementary Table S2).

Owing to limited assay availability, the first 1,110 randomized infants who completed the study were included in the immunogenicity analysis. From these, subjects with pre-existing IgA antibody titers of 20 U/mL or more before the first vaccine dose were eliminated to specifically assess vaccine-induced seroconversion. This exclusion applied to 12 participants. Additionally, subjects with missing immunization serological data in the laboratory report (*n* = 84), either due to unprocessed samples for limited assay capacity or reports of non-compliance with standard quality requirements, such as hemolysis, were also removed from the analysis. These exclusions did not significantly affect the balance of the study cohort’s group composition (Figure 1).

### Baseline data

The demographic characteristics of the infants enrolled in the seven study groups are presented in Table 1. The study comprised a total of 1,498 infants, with each group consisting of 214 participants. The demographic variables demonstrated a balanced distribution across all groups. Data is shown in Table 1.

### Immunogenicity

From the initial cohort of 1,498 infants, a subsample of 1,110 was selected due to assay constraints. This immunogenicity subsample comprised the first 1,110 infants who were enrolled and completed the study protocol. We confirmed no significant differences between the subsample and excluded infants in terms of sex distribution (*p* = 0.831) and gestational age (*p* = 0.768). The groups were evenly represented, and birth weights were similar (*p* = 0.832), although a significant difference in median age was observed (*p* < 0.001) (Supplementary Table S3).

The per-protocol immunogenicity analysis involved 1,014 infants’ data of post-immunity antibody titers. Age at enrollment was considered as a potential confounder. Nonetheless, no significant differences were found in age at enrollment across the seven study groups (median 52 days, *p* = 0.434) (Supplementary Table S4).

The aim of the immunogenicity analysis was to assess the non-inferiority of the mixed vaccine schedules compared to the standard single vaccine reference groups. The immunogenicity response was quantitatively evaluated by analyzing all groups’ geometric mean antibody titers. Group 1 (RV1 + RV1 + Placebo) and Group 2 (RV5 + RV5 + RV5) were used as reference standards. Groups 3 and 7 were compared with Group 1, while Groups 4, 5, and 6 were compared with Group 2.

**Figure 2:**
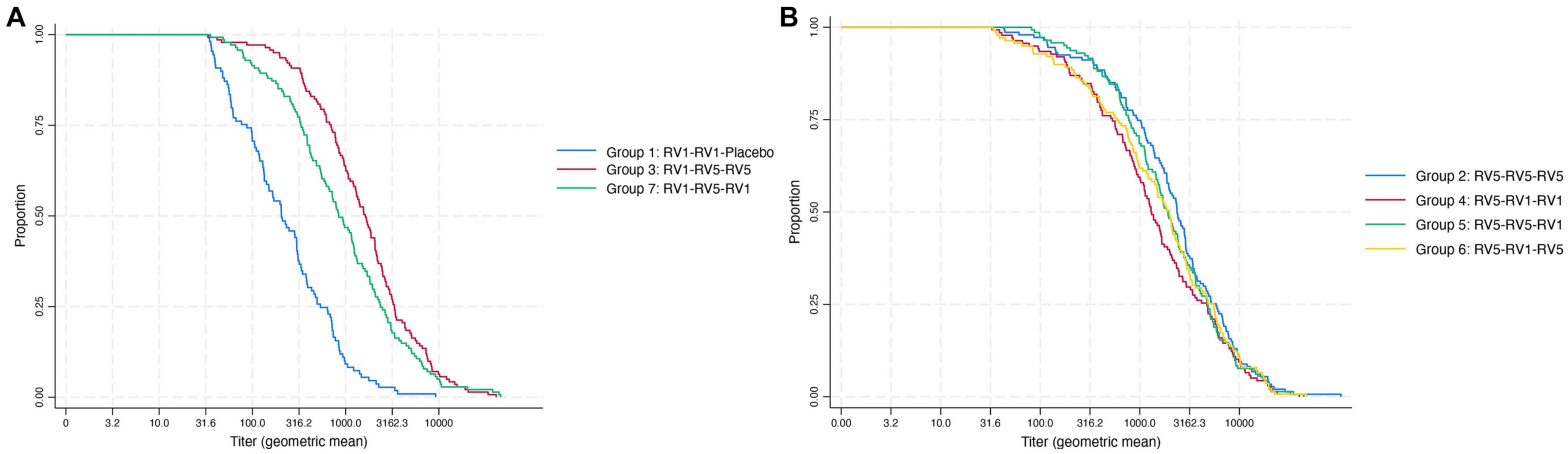
Immunogenicity response. Geometric mean titers reached post-immunization across the vaccine schedule. **(A)** Mixed schedules for group 3 (RV1 + RV5 + RV5) and group 7 (RV1 + RV5 + RV1) compared with group 1 (RV1 + RV1 + placebo) as reference group. **(B)** Mixed schedules for group 4 (RV5 + RV1 + RV1), group 5 (RV5 + RV5 + RV1), and group 6 (RV5 + RV1 + RV5) compared with group 2 (RV5 + RV5 + RV5) as the reference group.

Figure 2 illustrates the immunogenicity response for the vaccine schedules, depicting the proportion of subjects achieving different antibody titer levels (expressed as geometric mean) after vaccination. Panel A presents the curves of Group 1 (RV1 + RV1 + placebo), Group 3 (RV1 + RV5 + RV5) and Group 7 (RV1 + RV5 + RV1), with Group 3 showing the highest level of protection as indicated by its rightmost curve. Panel B shows the response curves for Group 2 (RV5 + RV5 + RV5), Group 4 (RV5 + RV5 + RV1), Group 5 (RV5 + RV5 + RV1), and Group 6 (RV5 + RV1 + RV5), with Group 2 exhibiting the highest protection levels among these groups.

**Figure 3:**
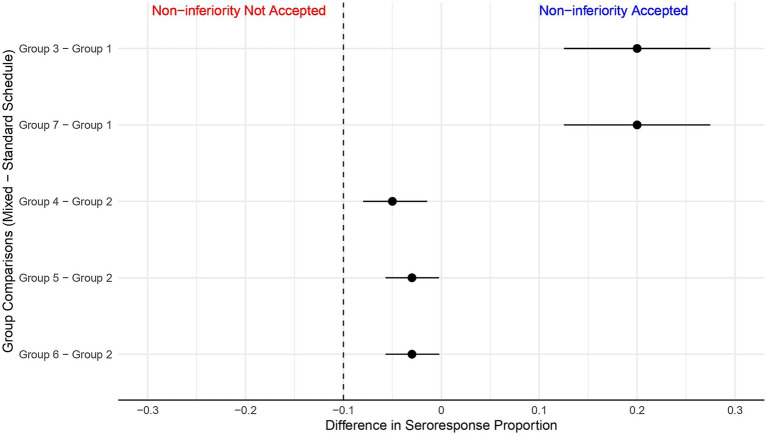
Noninferiority analysis. Differences in the proportion of seroresponding subjects. The predefined noninferiority threshold for comparison between each mixed schedule and the single vaccine regimens (RV1, RV5) was set at −0.1 (dashed vertical line). Noninferiority is accepted if the lower bound of the 95% confidence interval for the rate of seroresponders in each group does not surpass the threshold of −0.1. Group 3 and Group 7 were compared with Group 1 (RV1 + RV1 + Placebo). Group 4, Group 5 and Group 6 were compared with Group 2 (RV5 + RV5 + RV5).

Group 2 (RV5 + RV5 + RV5)2 exhibited the highest increase in geometric mean concentrations of antibodies, indicative of a strong immunogenic response, while Group 1 (RV1 + RV1 + placebo) had the smallest increase when compared to all other schedules (Table 2). In comparison with Group 1, Groups 3 and 7 demonstrated significant differences in the proportion of seroresponding subjects after vaccination, as indicated in Table 2. The seroresponse rate for mixed schedules in Groups 3 and 7 was identical (0.97). The difference in proportion of seroresponding subjects of these mixed schedules compared to the RV1 standard schedule in Group 1 was 0.20, with a 95% confidence interval (CI) for the difference ranging from 0.125 to 0.275, as shown in Figure 3.

**Table 2:** Immunogenicity response.

	GMC (95% CI)	*n*/N*	Proportion of subjects seroresponding (IgA ≥ 20 U/mL) (95% CI)
Group 1 RV1-RV1-Placebo	109.7 (89.7, 134.3)	109/141	0.77 (0.7005, 0.8439)
Group 3 RV1-RV5-RV5	576.97 (477.7, 696.8)	141/146	0.97^a^ (0.9423, 0.9976)
Group 7 RV1-RV5-RV1	369.52 (298.7,457.2)	141/146	0.97^a^ (0.9423, 0.9976)
Group 2 RV5-RV5-RV5	766.0 (627.5, 935.1)	147/147	1.00
Group 4 RV5-RV1-RV1	516.0 (412.9, 644.8)	137/144	0.95 (0.9144, 0.9855)
Group 5 RV5-RV5-RV1	692.8 (574.4, 835.7)	143/147	0.97 (0.9144, 0.9855)
Group 6 RV5-RV1-RV5	582.6 (462.3, 734.2)	139/143	0.97 (0.9424, 0.9975)
Total	473.7 (434.8, 515.9)	958/1014	0.94 (0.9258, 0.9546)

*n, number of seroresponding subjects; *N*, total number of participants per group; GMC, Geometric mean concentrations of antibody titers; 95% CI, 95% confidence interval.

a Difference of proportion test *p*-value < 0.001 vs. Group 1 (RV1-RV1-placebo).

The mixed vaccine schedules of Groups 4, 5, and 6 achieved proportions of seroresponders that were non-inferior to the reference Group 2 (RV5 standard schedule) as the differences were above the non-inferiority criteria in terms of eliciting an immunogenic response (Figure 3).

It is important to note that the observed differences were significantly positive, favoring the mixed schedule groups (Figure 3). This outcome supports mixed schedules’ interchangeability in eliciting an immunogenic response.

### Reactogenicity and safety

Our study’s safety profile was rigorously assessed across all seven groups, encompassing the entire vaccinated cohort. As depicted in Table 3, the reactogenicity-related symptoms—specifically diarrhea, vomiting, and fever—were recorded following each of the three vaccine doses administered.

**Table 3:** Proportion of subjects with reactogenicity-related symptoms in study groups.

Group	Diarrhea					Vomiting					Fever				
	Dose 1	Dose 2	Dose 3	Proportion	Difference	Dose 1	Dose 2	Dose 3	Proportion	Difference	Dose 1	Dose 2	Dose 3	Proportion	Difference
				(95% CI)	(95% CI)				(95% CI)	(95% CI)				(95% CI)	(95% CI)
Group 1	14/206	2/195	10/191	0.12	*Reference*	4/206	1/195	2/191	0.03	*Reference*	68/206	80/195	34/191	0.85	*Reference*
				(0.08,0.17)					(0.01,0.07)					(0.80,0.90)	
Group 3	3/206	4/198	9/198	0.07	−0.05*	0/206	3/198	4/198	0.03	0	62/210	81/198	47/198	0.96	0.11*
				(0.04,0.11)	(−0.10,−0.009)				(0.01,0.07)	(−0.03,0.03)				(0.92–0.98)	(0.05,0.16)
Group 7	7/207	9/200	9/197	0.12	-0.009	1/207	5/200	3/197	0.03	0	70/207	82/200	43/197	0.91	0.06
				(0.08,0.17)	(−0.07,0.05)				(0.01,0.07)	(−0.03,0.03)				(0.86–9.95)	(‘-0.0004,0.12)
Group 2	8/210	6/204	13/201	0.13	*Reference*	3/210	1/204	3/201	0.03	*Reference*	65/207	86/204	46/200	0.91	*Reference*
				(0.08,0.18)					(0.01,0.07)					(0.86,0.94)	
Group 4	4/208	5/198	5/196	0.07	−0.06*	1/208	2/198	3/196	0.03	−0.005	65/207	85/198	42/196	0.90	−0.009
				(0.04,0.11)	(−0.12,-0.005)				(0.01,0.06)	(−0.04,0.03)				(0.85,0.93)	(‘-0.07, 0.05)
Group 5	6/209	7/199	6/197	0.09	−0.04	0/209	1/199	2/197	0.01	−0.02	58/209	79/199	37/197	0.82	−0.09*
				(0.05,0.14)	(−0.09,0.02)				(0.003,0.04)	(−0.05,0.01)				(0.76,0.87)	(−0.15,−0.02)
Group 6	7/207	5/200	11/198	0.11	-0.02	3/208	1/200	1/198	0.02	−0.009	76/208	98/200	36/198	0.98	0.075*
				(0.07,0.16)	(−0.08,0.04)				(0.008,0.05)	(−0.04,0.02)				(0.95,0.99)	(0.03,0.12)
Total	49/1453	38/1394	63/1378	0.08		12/1454	14/1394	18/1378	0.03		476/1453	591/1394	285/1377	0.94	
				(0.06,0.09)					(0.02,0.04)					(0.93,0.96)	

*Difference of proportion test *p*-value < 0.05. Pairwise comparisons: Groups 3 and 7 are compared with Group 1; Groups 4, 5, and 6 are compared with Group 2.

Notably, fever was the most frequently reported symptom post-vaccination, but the occurrences remained within the expected ranges for pediatric vaccinations.

The proportion of subjects with fever at any grade (>38°C) was higher in Group 3 compared to Group 1. However, this increase did not translate into a higher rate of hospitalization, which remained exceedingly low across all groups.

No increases were detected in the incidence of any severity of diarrhea and vomiting across the mixed groups compared with the standard schedules (Table 3).

During the study, there were only ten hospitalization events reported, with none attributed to the vaccination regimen (Table 4). Following the first dose, there were three hospitalizations: two cases of bronchiolitis and one for a planned inguinal hernioplasty. After the second dose, there were seven hospitalizations due the bronchiolitis (1 case), pyelonephritis (2 cases), and community-acquired pneumonia (4 cases), one of which involved septic shock. No hospitalizations were reported following the third dose. Additionally, our study did not record any instances of intussusception or deaths. This underscores the tolerability and safety of the vaccine schedules under investigation.

**Table 4:** Proportion of subjects with hospitalization events reported during the study by groups.

Group	Hospitalization				
	Dose 1	Dose 2	Dose 3	Proportion	Difference
				(95% CI)	(95% CI)
Group 1	0/205	2/194	0/190	0.010	*Reference*
				(0.001, 0.03)	
Group 3	0/206	2/198	0/198	0.010	0
				(0.001, 0.03)	(−0.02, 0.02)
Group 7	2/207	1/200	0/198	0.014	0.004
				(0.003, 0.04)	(−0.02, 0.02)
Group 2	0/210	1/204	0/201	0.005	*Reference*
				(0.0001, 0.03)	
Group 4	1/207	0/198	0/196	0.005	0
				(0.0001, 0.03)	(−0.013, 0.013)
Group 5	0/209	0/199	0/197	0	−0.006
				(0.0, 0.02)	(−0.02, 0.006)
Group 6	0/208	1/200	0/198	0.005	0
				(0.0001, 0.03)	(−0.013, 0.013)
Total	3/1452	7/1393	0/1378	0.007	
				(0.003, 0.01)	

## Discussion

After its initial recommendation in 2006, the World Health Organization (WHO) reiterated in 2009 the crucial need to provide rotavirus vaccinations to infants globally. Additionally, in 2008, the Advisory Committee on Immunization Practices (ACIP) suggested that children who could not receive the same type of licensed rotavirus vaccine for follow-up doses should instead receive three doses comprising different vaccine types (35).

The cost of licensed vaccines and logistic challenges pose significant concerns. These factors could be deterrents to widespread availability in public health systems of many developing countries, where infants face a heavier burden of rotavirus-related morbidity and mortality compared to their counterparts in developed, high-income countries (36).

Extensive scientific evidence has established the efficacy of rotavirus vaccines RV5 (RotaTeq™) and RV1 (Rotarix™), which provide lasting protection against various strains, including those most predominantly circulating (37). Their efficacy extends to various clinical outcomes, including all-cause diarrhea, acute and severe gastroenteritis, and both any rotavirus-specific gastroenteritis and severe rotavirus-specific gastroenteritis. Additionally, these vaccines have been shown to reduce hospitalizations due to any cause of gastroenteritis and severe rotavirus gastroenteritis, maintaining sustained efficacy over one or more years post-vaccination. The efficacy data is supported by randomized controlled trials conducted in low- and high-mortality countries, comparing rotavirus vaccine schedules (RV1 or RV5) with a placebo (38, 39). Heterogeneity among studies is often attributed to differences in definitions of outcomes as severe illnesses. Notably, of head-to-head studies between RV1 and RV5 are lacking.

A Bayesian network meta-analysis, which reevaluated data from a 2012 Cochrane Review, addressed the indirect comparison of RV1 and RV5 effectiveness. This study found no significant differences in effectiveness between RV1 and RV5 in preventing severe rotavirus diseases up to two years (OR 2.23, 95%CI 0.71–5.20) (40). However, the wide credible interval reported underscores the need for ongoing research, ideally including controlled trials for direct vaccine comparisons and updated data collection, particularly in regions with varying income strata and child mortality rates.

After the introduction of these vaccines, variations in their efficacy have led to observable differences in vaccine effectiveness, which also seem to be context-dependent (41). Meta-analyses highlight a performance gradient in vaccine effectiveness, influenced by child mortality rates, with better outcomes in lower mortality settings (42). The varied levels of protection observed across different settings are acknowledged to be multifactorial. Factors such as concurrent administration of live oral polio vaccine, bacterial or viral infections, nutritional status, microbiome composition, and maternal antibodies potentially lead to reduced immunogenicity (13, 41, 43).

Moreover, the protective effect of vaccines is recognized as dose-dependent (16). However, ensuring consistent access to vaccines from the same manufacturer for each dose in a standard schedule may not always be feasible due to potential vaccine shortages after administering one or two doses. This uncertainty poses a significant concern in attaining adequate immune responses. Therefore, assessing the immune response to mixed vaccine schedules involving vaccines with different characteristics and dosing requirements is essential.

Over the years since the introduction of rotavirus vaccines, intestinal IgA response and its surrogate, serum IgA, have long been considered the principal correlates of protection for rotavirus vaccines (34, 44). A serum IgA level exceeding 20 U/mL is often cited as protective (32). A correlation has been reported between serum anti-rotavirus IgA antibody titers and protection against clinical rotavirus-related outcomes, although serum IgA itself is not the sole immunological effector of this protection (33).

Our study demonstrates that vaccination with combined dosing induces immune responses that are non-inferior to those elicited by complete RV5 or RV1 schedules. Additionally, RVI and RV5 were administered along with other routinely administered pediatric vaccines, thus allowing for safety and immunogenicity to be assessed as the mixed schedules would be administered in routine use.

This study demonstrates seroconversion rates for RVI and RV5 schedules that are comparable to those reported in a clinical trial performed in a high-income country (United States). In that context, seroconversion frequencies based on anti-rotavirus IgA were found to be 0.77 (95%IC: 0.72–0.82) for RV1 and 0.91 (95%IC, 0.87–9.95) for RV5 (23). The comparison of seroresponse rates between our mixed and standard schedules groups revealed that the immune response induced by the mixed schemes was not-inferior to standard schemes, as the observed differences in seroconversion rates did not exceed the non-inferiority predefined margin of −0.10. This margin is an accepted criterion for vaccine schedule comparisons (20, 23, 24).

After vaccination, 94% of infants had titters higher than 20 U/mL. Although GMCs for Group 1 were lower than those of the other groups, the GMC antibodies were 5.4-fold higher than the 20 U/mL threshold.

A high proportion of infants tested positive for antibodies (≥ 20 U/mL) in all study groups, with mixed vaccine schedules deemed non-inferior to the standard schedules using RV5 and RV1. The percentage of seropositive infants was higher in Group 2 (standard RV5 schedule). In contrast, Group 1 (standard RV1 schedule) exhibited lower immunogenicity parameters. It’s important to note that Group 1 effectively received only two doses of the vaccine, as the third was a placebo. However, the seroresponse rate in the RV1 standard group was 0.77, aligning with previous studies (23, 45, 46). The observed equivalency in immunogenicity among groups receiving mixed schedules of RV1 and RV5 further supports the relevance of completing the schedules with the available vaccine.

Rotavirus-specific IgA is a surrogate marker of protection rather than an absolute correlate (47). Its association with protection varies across populations. Baker et al. (34) found that higher post-immunization rotavirus-specific IgA levels were associated with a reduced incidence of severe rotavirus gastroenteritis, with more pronounced protection in low child-mortality countries compared to high child-mortality I.

Research in middle-income settings like Mexico indicates an association between higher serum RV-IgA levels and reduced risk of rotavirus infection, diarrhea, and moderate-to-severe gastroenteritis (48, 49).

The evidence shows that in low-income countries this link appears less convincing (33, 41). Lee et al. observed that post-immunization rotavirus-specific IgA was not an optimal correlate of protection against rotavirus gastroenteritis among infants in Bangladesh, where the efficacy of rotavirus vaccines is around 50% (50). However, a study in Malawi, a country with a high rotavirus burden, indicates that low serum RV-specific IgA is associated with a greater risk of vaccine failure (47).

This suggests that while serum IgA is a valuable marker of vaccine immunogenicity, it may not fully encompass the complexity of the vaccine-induced immune response, nor is it necessarily a direct causative factor in protection (41). The immediate individual impact of vaccine interchangeability, particularly for already effective vaccines, is often assessed based on comparative immunogenicity, reactogenicity, and safety profiles (20, 23).

The relevance of imperfect immunological correlates increases as conducting extensive efficacy trials becomes more challenging (41). In the context of planned equivalence clinical trials featuring randomized designs to minimize potential confounders, measuring serological correlates within a defined 4–6-week window post-vaccination is useful for discerning seroresponse profiles following mixed schemes of licensed vaccines. This step is essential in the short term. Future efficacy studies that integrate data on rotavirus-specific IgA, along with additional measurement of candidate serological correlates and RV-specific cellular parameters at the onset of rotavirus gastroenteritis (47), could provide valuable insights into the field of vaccine interchangeability.

Previous studies have shed light on the immunogenicity of mixed rotavirus vaccine schedules. A notable study in the United States investigated the noninferiority of immune responses to mixed schedules of RV1 and RV5 vaccines compared to single-vaccine regimens. The study examined three mixed schedules (RV5-RV1-RV1, RV5-RV1-RV1, and RV1-RV5-RV5), revealing that the proportion of children seropositive to at least one vaccine antigen ranged from 77 to 96%, with no significant differences across groups (23).

A secondary analysis of surveillance data in the USA involving 2,425 children indicated that 75 (3.1%) received a complete 3-dose rotavirus vaccination regimen with mixed RV5 and RV1 vaccines. These mixed schedules retained a statistically significant level of protection (80%) against rotavirus gastroenteritis, like that observed for complete, single vaccine–type schedules.

A case–control study in an urban, southeastern USA population reported that children with complete rotavirus vaccination, whether standard or mixed RV1 or RV5, were protected against rotavirus (complete mixed: OR 0.29, 95%CI 0.12–0.72; and complete RV5 and RV1: OR 0.21, 95%CI 0.14–0.31). Specific combinations of doses were not declared. Notably, children who received a complete mixed schedule did not exhibit significantly higher protection against rotavirus test-positive disease compared to unvaccinated children when factors such as age, race, ethnicity, insurance status, and disease severity were controlled. This finding was attributed to the small sample size of children who received complete mixed vaccine (*n* = 27, 4.0%). The authors noted that that incomplete vaccination schedules were largely due to the availability of RV1 and RV5 vaccines from manufacturers (45).

The complete 3-dose schedule for the RV5 exhibits logistical challenges due to its requirement for more doses and a larger volume per dose compared to RV1. This necessitates more extensive cold-chain resources, which can impact its availability. Therefore, our design did not consider the inclusion of mixed schedules with the combination of RV1 and RV5 at two-dose series; this point is relevant to be able to conclude the possibility of reaching the immunogenicity goal with shorter mixed schedules (16). This is a perspective to probe strategies that increase flexibility while being evidence-based.

New vaccines have been developed and approved in some Asian countries, and even more, they have received WHO pre-qualification (RotaSIIL™ and ROTAVAC™). However, these vaccines are not available in Mexico. These vaccines are possibly lower cost vs. the studied RV5/RV1 options that might need to be considered for rotavirus prevention in the future (51). Interestingly, a clinical trial at two sites in India recently reported interchangeability in terms of immunogenicity between ROTAVAC™ and RotaSIIL™. The safety profile was similar across the study groups (20).

Our study also demonstrates that RV1 and RV5 are safe when administered interchangeably. The safety assessment across all groups showed comparable reactogenicity profiles, with no new safety concerns detected. The overall incidence of fever, vomiting, and diarrhea was similar across groups, aligning with the established safety profiles of RV1 and RV5. The slightly higher incidence of fever in some groups, potentially influenced by concurrent vaccinations, did not translate into increased hospitalization rates or other serious adverse events.

Our study found that the total proportion of diarrhea was 0.08 (95% confidence interval 0.06–0.09). We also observed a slightly higher incidence of diarrhea related to vaccination in group 1, who received the complete RV1 schedule. The proportion of diarrhea in this group was 0.12, and the highest incidence was observed after the first dose, dropping to a single report after the second dose. Unexpectedly, diarrhea reporting increased after the placebo was used in this group. A previous study found similar incidence rates of approximately 0.14 for both the Rotarix™ vaccine and the placebo (52). The proportion of diarrhea was comparable across all vaccine groups and was consistent with other studies based on mixed RV1 and RV5 schedules (23).

Our study did not report any vaccine-related hospitalization events nor identify any cases of intussusception, the rare adverse event historically associated with rotavirus vaccines (53). The adherence to a rigorous protocol comprehensive active monitoring of adverse events, and our intention-to-treat analysis form a strong foundation for our findings on reactogenicity and safety. This supports these vaccines’ safety when administered in mixed and standard schedules.

The findings from our study align with the emerging consensus on the comparable safety profile of these vaccines in different settings (40, 54) and underscore the potential for flexible vaccination strategies, especially in settings where vaccine availability may fluctuate.

This study contributes valuable insights into rotavirus vaccine immunogenicity and safety profiles using mixed schedules. Our results, in conjunction with the body of evidence already published, support the Advisory Committee in Immunization Practice (ACIP) recommendations to complete rotavirus vaccination schedules in children. The safety profile observed is adequate, and the achieving immunogenicity is promising.

However, we acknowledge certain limitations in our study. Our study cohort’s demographic and geographic specificity may limit our findings’ external validity or generalizability. Although we achieved a high retention rate of participants in the clinical follow-up (>87%), the limited availability of reagents for immunogenicity testing was a significant constraint. Approximately 75% of those attending post-vaccination visits were included in the immunogenicity analysis across all groups, a factor that should be considered when interpreting our results. Additionally, the study had immunogenicity in the short term as the primary outcome and did not incorporate rotavirus-related gastroenteritis and other clinical outcomes.

A key area for future research is to assess whether these findings are consistently reflected in vaccine efficacy, particularly exploring schedules that might yield the most favorable clinical outcomes. These findings provide evidence to inform decision-makers and healthcare providers in the implementation of adaptable and effective rotavirus vaccination strategies.

## Conclusion

Mixed rotavirus vaccine schedules have been demonstrated to be safe and non-inferior in eliciting immune responses compared to standard schedules using a single formulation of licensed rotavirus vaccines. This study offers valuable insights into the interchangeability and selection of rotavirus vaccines, thereby contributing significantly to the enhancement of global rotavirus vaccination strategies. Particularly in scenarios of vaccine shortages, these findings underscore the utility of mixed vaccine schedules in preventing rotavirus diarrhea. Such flexibility in vaccination approaches could be crucial in ensuring broader and more effective pediatric coverage against rotavirus globally.

## Data availability statement

The raw data supporting the conclusions of this article will be made available by the authors, without undue reservation.

## Ethics statement

The study was conducted in accordance with the Declaration of Helsinki and the International Conference on Harmonization’s Good Clinical Practice guidelines. The study protocol was reviewed and approved by the Ethics Research Committee of the Instituto Nacional de Pediatría (reference number CE/675/2012). Additionally, the study was authorized by the Health Authorization Commission as part of the Federal Commission for Protection against Health Risks (COFEPRIS), under authorization number INP-15-2012. All procedures were conducted under the applicable local legislation and institutional requirements. Parents or legally acceptable representatives of the infants provided their written informed consent prior to enrollment in the study. This study was registered in ClinicalTrials.gov on July 17, 2014, under the identifier NCT02193061, with the protocol name: ‘Randomized, Controlled Single-blind Clinical Study to Assess Vaccine Interchangeability Between RV5 and RV1 Using Seven Combined Anti-Rotavirus Prevention Programs’. The CONSORT 2010 checklist of information to include when reporting a randomized trial is provided in the Supplementary material (Supplementary Table 5).

## Author contributions

MM-P: Writing – original draft, Writing – review & editing, Methodology, Conceptualization, Funding acquisition, Resources. PV-V: Conceptualization, Data curation, Formal analysis, Methodology, Validation, Writing – review & editing. JR-F: Conceptualization, Methodology, Writing – original draft. MR-W: Investigation, Project administration, Supervision, Writing – original draft. HM-M: Data curation, Formal analysis, Visualization, Writing – original draft. IH-B: Investigation, Supervision, Writing – original draft. SF-G: Validation, Writing – original draft. VLR: Conceptualization, Funding acquisition, Writing – review & editing. PV-C: Data curation, Formal analysis, Methodology, Validation, Visualization, Writing – review & editing.
