# Supplementary material for: Immunogenicity of RV1 and RV5 vaccines administered in standard and interchangeable mixed schedules: a randomized, double-blind, non-inferiority clinical trial in Mexican infants
Source: Front Public Health. 2024 Feb 23;12:1356932. doi: 10.3389/fpubh.2024.1356932 (PMC10920348; doi:10.3389/fpubh.2024.1356932)
Supplement: Supplementary file 1 [file Data_Sheet_1.docx]

Immunogenicity of RV1 and RV5 Vaccines Administered in Standard and Interchangeable Mixed Schedules: A Randomized, Double-blind, Non-Inferiority Clinical Trial in Mexican Infants

Supplementary Material

# Supplementary Tables

**Supplementary Table 1.** Vaccine Schedules

| Vaccine Schedule Group | 2 months old (± 2 weeks) | 2 months later (53 to 67 days) | 2 months later (53 to 67 days) |
| --- | --- | --- | --- |
| Group 1 | RV1 | RV1 | Placebo |
| Group 2 | RV5 | RV5 | RV5 |
| Group 3 | RV1 | RV5 | RV5 |
| Group 4 | RV5 | RV1 | RV1 |
| Group 5 | RV5 | RV5 | RV1 |
| Group 6 | RV5 | RV1 | RV5 |
| Group 7 | RV1 | RV5 | RV1 |

RV1 = Monovalent vaccine (Rotarix^TM^), RV5 = Pentavalent vaccine (RotaTeq^TM^)

**Supplementary Table 2.** Reasons for subject’s discontinuation or elimination

|  | Group 1  n=24 | Group 2  n=16 | Group 3  n=17 | Group 4  n=21 | Group 5  n=23 | Group 6  n=16 | Group 7  n=18 |
| --- | --- | --- | --- | --- | --- | --- | --- |
| Lost to follow-up | 13 | 10 | 11 | 12 | 17 | 11 | 13 |
| Withdrawal by parent/guardian | 7 | 3 | 4 | 4 | 2 | 2 | 3 |
| Other illness/non-study medical interventions | 1 | 0 | 0 | 1 | 0 | 0 | 0 |
| Protocol deviations | 1 | 1 | 1 | 1 | 0 | 1 | 0 |
| Sample loss |  |  |  |  |  |  |  |
| Venipuncture failure | 0 | 0 | 1 | 1 | 2 | 0 | 1 |
| Sample coagulation | 2 | 2 | 0 | 2 | 2 | 2 | 0 |
| Insufficient sample volumen | 0 | 0 | 0 | 0 | 0 | 0 | 1 |

**Supplementary Table 3.** Comparison between immunity analysis subsample subjects and subjects not included.

|  | Eliminated | Immunity analysis subsample | p-value* |
| --- | --- | --- | --- |
| N = 1498 | 388 (25.9%) | 1,110 (74.1%) |  |
| Sex |  |  |  |
| Male | 194 (50.0%) | 548 (49.4%) | 0.831 |
| Female | 194 (50.0%) | 562 (50.6%) |  |
| Group |  |  |  |
| 1 | 56 (14.4%) | 158 (14.2%) | 1.000 |
| 2 | 55 (14.2%) | 159 (14.3%) |  |
| 3 | 55 (14.2%) | 159 (14.3%) |  |
| 4 | 56 (14.4%) | 158 (14.2%) |  |
| 5 | 55 (14.2%) | 159 (14.3%) |  |
| 6 | 55 (14.2%) | 159 (14.3%) |  |
| 7 | 56 (14.4%) | 158 (14.2%) |  |
| Age, days | 58 (50-63) | 52 (46 -60) | <0.001 |
| Gestational age, weeks | 39 (38-40) | 39 (38-40) | 0.768 |
| Birth weight, kg | 3052.5 (2817-3340) | 3075 (2800-3375) | 0.832 |

* Pearson test for sex and treatment group and Kruskal-Wallis test across characteristics of patients with samples included to immunogenicity subsample *vs.* eliminated samples

**Supplementary Table 4.** Characteristics of the study groups for the per-protocol immunogenicity analysis involved 1,014 infants.

|  | Group | | | | | | | |
| --- | --- | --- | --- | --- | --- | --- | --- | --- |
|  | 1 | 2 | 3 | 4 | 5 | 6 | 7 | p-value* |
| N | 141 (13.9%) | 147 (14.5%) | 146 (14.4%) | 144 (14.2%) | 147 (14.5%) | 143 (14.1%) | 146 (14.4%) |  |
| Sex |  |  |  |  |  |  |  |  |
| Male | 70 (50.4%) | 67 (45.3%) | 76 (52.1%) | 75 (51.7%) | 69 (46.9%) | 70 (49.0%) | 71 (48.6%) | 0.905 |
| Female | 69 (49.6%) | 81 (54.7%) | 70 (47.9%) | 70 (48.3%) | 78 (53.1%) | 73 (51.0%) | 75 (51.4%) |  |
| Age | 52 (47-59) | 50 (45-59) | 52.5 (46-60) | 52 (47 -60) | 53 (46-60) | 51 (46-60) | 52.5 (46-60) | 0.434 |
| Gestational age (weeks) | 39 (38-40) | 39 38(38-40) | 39 (38-40) | 39 (38-40) | 39 (38-40) | 39.5 (38-40) | 39.5 (38-40) | 0.406 |
| Birth weight (g) | 3125  (2770-3400) | 3045  (2790-3361.5) | 3052  (2820-3340) | 3132.5  (2820-3440) | 3035  (2785-3315) | 3040  (2755-3350) | 3047  (2790-3355) | 0.806 |

*Pearson test across levels of the group for sex and Kruskal Wallis test across levels of the group for age, gestational age, and birth weight.

Supplementary Table 5. CONSORT checklist.


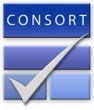
CONSORT 2010 checklist of information to include when reporting a randomized trial*

| Section/Topic | Item No | Checklist item | Reported |
| --- | --- | --- | --- |
| Title and abstract | | | |
|  | 1a | Identification as a randomized trial in the title [identification as a noninferiority randomized trial] | √ |
|  | 1b | Structured summary of trial design, methods, results, and conclusions | √ |
| Introduction | | | |
| Background and objectives | 2a | Scientific background and explanation of rationale [rationale for using a noninferiority design] | √ |
|  | 2b | Specific objectives or hypotheses [hypotheses concerning noninferiority, specifying the noninferiority margin with the rationale for this choice] | √ |
| Methods | | | |
| Trial design | 3a | Description of trial design (such as parallel, factorial) including allocation ratio | √ |
|  | 3b | Important changes to methods after trial commencement (such as eligibility criteria), with reasons | N⁄A |
| Participants | 4a | Eligibility criteria for participants [whether participants in the noninferiority trial are similar to those in any trial that established efficacy of the reference statement] | √ |
|  | 4b | Settings and locations where the data were collected | √ |
| Interventions | 5 | The interventions for each group with sufficient details to allow replication, including how and when they were actually administered [whether the reference treatment in the noninferiority trial is identical (or very similar) to that in any trial(s) that established efficacy] | √ |
| Outcomes | 6a | Completely defined pre-specified primary and secondary outcome measures, including how and when they were assessed [specify the noninferiority outcomes and whether hypotheses for main and secondary outcomes are noninferiority] | √ |
|  | 6b | Any changes to trial outcomes after the trial commenced, with reasons | N/A |
| Sample size | 7a | How sample size was determined [whether the sample size was calculated using a noninferiority criterion and what the noninferiority margin was] | √ |
|  | 7b | When applicable, explanation of any interim analyses and stopping guidelines [to which outcomes they apply and whether related to a noninferiority hypothesis] | N/A |
| Randomization: |  |  |  |
| Sequence generation | 8a | Method used to generate the random allocation sequence | √ |
|  | 8b | Type of randomization; details of any restriction (such as blocking and block size) | √ |
| Allocation concealment mechanism | 9 | Mechanism used to implement the random allocation sequence (such as sequentially numbered containers), describing any steps taken to conceal the sequence until interventions were assigned | √ |
| Implementation | 10 | Who generated the random allocation sequence, who enrolled participants, and who assigned participants to interventions | √ |
| Blinding | 11a | If done, who was blinded after assignment to interventions (for example, participants, care providers, those assessing outcomes) and how | √ |
|  | 11b | If relevant, description of the similarity of interventions | N/A |
| Statistical methods | 12a | Statistical methods used to compare groups for primary and secondary outcomes [whether a 1- or 2-sided confidence interval approach was used] | √ |
|  | 12b | Methods for additional analyses, such as subgroup analyses and adjusted analyses | √ |
| Results | | | |
| Participant flow (a diagram is strongly recommended) | 13a | For each group, the numbers of participants who were randomly assigned, received intended treatment, and were analyzed for the primary outcome | √ |
|  | 13b | For each group, losses, and exclusions after randomization, together with reasons | √ |
| Recruitment | 14a | Dates defining the periods of recruitment and follow-up | √ |
|  | 14b | Why the trial ended or was stopped | N/A |
| Baseline data | 15 | A table showing baseline demographic and clinical characteristics for each group | √ |
| Numbers analyzed | 16 | For each group, number of participants (denominator) included in each analysis and whether the analysis was by original assigned groups | √ |
| Outcomes and estimation | 17a | For each primary and secondary outcome, results for each group, and the estimated effect size and its precision (such as 95% confidence interval) [for the outcome for which noninferiority was hypothesized a figure showing confidence intervals and the noninferiority margin may be useful] | √ |
|  | 17b | For binary outcomes, presentation of both absolute and relative effect sizes is recommended | √ |
| Ancillary analyses | 18 | Results of any other analyses performed, including subgroup analyses and adjusted analyses, distinguishing pre-specified from exploratory | N/A |
| Harms | 19 | All important harms or unintended effects in each group | N/A |
| Discussion | | | |
| Limitations | 20 | Trial limitations, addressing sources of potential bias, imprecision, and, if relevant, multiplicity of analyses | √ |
| Generalizability | 21 | Generalizability (external validity, applicability) of the trial findings | √ |
| Interpretation | 22 | Interpretation consistent with results, balancing benefits, and harms, and considering other relevant evidence [Interpret results in relation to the noninferiority hypothesis] | √ |
| Other information | | | √ |
| Registration | 23 | Registration number and name of trial registry | √ |
| Protocol | 24 | Where the full trial protocol can be accessed, if available | √ |
| Funding | 25 | Sources of funding and other support (such as supply of drugs), role of funders | √ |

* [www.consort-statement.org](http://www.consort-statement.org).
CONSORT (Consolidated Standards of Reporting Trials) Statement, extended recommendations to noninferiority and equivalence trials are enclosed in brackets. N/A: Not Applicable, √ included in the manuscript.
